# ART use and associated factors among HIV positive caregivers of orphans and vulnerable children in Tanzania

**DOI:** 10.1186/s12889-020-09361-6

**Published:** 2020-08-17

**Authors:** Amon Exavery, John Charles, Asheri Barankena, Erica Kuhlik, Godfrey M. Mubyazi, Kassimu Tani, Amal Ally, Epifania Minja, Alison Koler, Levina Kikoyo, Elizabeth Jere

**Affiliations:** 1 Pact, P.O. Box 6348, Dar es Salaam, Tanzania; 2 Pact, Inc., 1828 L St NW Suite 300, Washington, DC, 20036 USA; 3 National Institute for Medical Research (NIMR), P.O Box 9653, Dar es Salaam, Tanzania

**Keywords:** Utilization, Antiretroviral therapy, HIV, Caregivers of orphans and vulnerable children, Kizazi Kipya, Tanzania

## Abstract

**Background:**

Utilization of antiretroviral therapy (ART) is crucial for better health outcomes among people living with the human immunodeficiency virus (PLHIV). Nearly 30% of the 1.6 million PLHIV in Tanzania are not on treatment. Since HIV positive status is the only eligibility criterion for ART use, it is critical to understand the obstacles to ART access and uptake to reach universal coverage of ART among PLHIV. For the caregivers of orphans and vulnerable children (OVC) LHIV and not on ART, attempts to identify them and ensure that they initiate and continue using ART is critical for their wellbeing and their ability to care for their children.

**Methods:**

Data are from the community-based, United States Agency for International Development (USAID)-funded Kizazi Kipya project that aims at scaling up the uptake of HIV/AIDS and other health and social services by orphans and vulnerable children (OVC) and their caregivers. HIV positive caregivers of OVC who were enrolled in the USAID Kizazi Kipya project between January 2017 and June 2018 were included in this cross-sectional study. The caregivers were drawn from 11 regions: Arusha, Iringa, Katavi, Kigoma, Mara, Mbeya, Morogoro, Ruvuma, Simiyu, Singida, and Tanga. The outcome variable was ART status (either using or not), which was enquired of each OVC caregiver LHIV at enrollment. Data analysis involved multivariable analysis using random-effects logistic regression to identify correlates of ART use.

**Results:**

In total, 74,999 caregivers living with HIV with mean age of 44.4 years were analyzed. Of these, 96.4% were currently on ART at enrollment. In the multivariable analysis, ART use was 30% lower in urban than in rural areas (adjusted odds ratio (OR) = 0.70, 95% confidence interval (CI) 0.61–0.81). Food security improved the odds of being on ART (OR = 1.29, 95% CI 1.15–1.45). Disabled caregivers were 42% less likely than non-disabled ones to be on ART (OR = 0.58, 95% CI 0.45–0.76). Male caregivers with health insurance were 43% more likely than uninsured male caregivers to be on ART (OR = 1.43, 95% CI 1.11–1.83). Caregivers aged 40–49 years had 18% higher likelihood of being on ART than the youngest ones. Primary education level was associated with 26% increased odds of being on ART than no education (OR = 1.26, 95% CI 1.13–1.41).

**Conclusions:**

Although nearly all the caregivers LHIV in the current study were on ART (96.4%), more efforts are needed to achieve universal coverage. The unreached segments of the population LHIV, even if small, may lead to worse health outcomes, and also spur further spread of the HIV epidemic due to unachieved viral suppression. Targeting caregivers in urban areas, food insecure households, who are uninsured, and those with mental or physical disability can improve ART coverage among caregivers LHIV.

## Background

In 2018, the global estimate of the number of people living with HIV was 37.9 million, 62% (23.3 million) of whom were accessing antiretroviral therapy (ART) [1]. Corresponding estimates of people living with HIV (PLHIV) were 20.6 million and 67% (13.8 million) on ART in Eastern and Southern Africa together [2]. In Tanzania, 1.6 million people were living with HIV in 2018, 71% of whom were accessing ART [3]. The use of ART by people living with HIV (PLHIV) is crucial for better health outcomes: ART decreases HIV-associated morbidity and mortality, as well as the incidence of new HIV infections [4].

In 2016, the World Health Organization (WHO) removed all barriers for ART eligibility, and recommended that all PLHIV should start ART early after undergoing confirmatory test for HIV (‘Test and Treat’), regardless of the WHO clinical stage or CD4 count [5]. The early use of ART keeps PLHIV alive and healthier, and ART significantly reduces the risk of onward transmission of HIV, as shown in a real-world setting in sub-Saharan Africa [6]. However, evidence shows that not all PLHV are on treatment, including those who know that they are infected with HIV [1–3, 7]. This suggests that barriers to ART use among PLHIV remain, necessitating further research and programming to address them and achieve universal ART coverage.

Tanzania introduced an ART program for HIV care and treatment in 2004. Since then, HIV/AIDS-related morbidity and mortality have been declining, quality of life of PLHIV improved, and the majority of PLHIV have resumed normal life [8]. The Government of Tanzania (GoT) in collaboration with development partners and civil society organizations (CSOs) has continued to scale up HIV/AIDS care and treatment services. As of 2017, up to 82.1% of all 7494 health facilities in the country offered care and treatment services to PLHIV. The GoT has also been working tirelessly to increase human resources as well as medical supplies and commodities. Furthermore, the GoT improves retention and adherence to ART through community engagement using community volunteers to support care and treatment services and minimizing loss to follow up (LTFU). In this case, more than 350 treatment advocates, 1002 community action teams and 213 cluster coordination teams have been formed and trained to support PLHIV on care [8].

Accordingly, the goals of the Tanzania Health Sector HIV and AIDS Strategic Plan IV, 2017–2022 (HSHSP IV) Monitoring and Evaluation Plan are geared to maximizing efforts and coverage of HIV prevention and treatment services, particularly for key and vulnerable populations [9]. These populations, according to the national HIV/AIDS response, are Female Sex Workers (FSW), People who Inject Drugs (PWID), Men who have Sex with Men (MSM), Adolescents Girls and Young Women (AGYW), fishers and fishing community, miners, agricultural plantations workers, long distance truck drivers, construction workers and students in higher learning institutions [8]. While individual caregivers of orphans and vulnerable children (OVC) may fall into one of these categories, as a group they are not identified as a key and vulnerable population despite their HIV risk factors and related health, social, and economic challenges in caring for OVC.

In 2014, the UNAIDS proposed the 90–90–90 targets, which called for a scale–up of HIV testing, so that by 2020, 90% of all PLHIV are aware of their status; 90% of all people with diagnosed HIV infection receive sustained ART; and 90% of all people receiving ART achieve viral suppression [4]. This analysis builds off the second 90, whereby, although data on HIV burden and ART coverage among OVC caregivers does not yet exist, in the general adult PLHIV population (age 15 years and above) in Tanzania, HIV prevalence is 4.7% and ART use among those who know their HIV positive status is 93.6% [10]. However, looking at ART coverage among all PLHIV in Tanzania, only 71% were on treatment at the end of 2018 [3]. Although the country has made considerable progress towards achievement of the UNAIDS 90–90-90 targets in adults [10], further efforts are needed to get all PLHIV on ART because the future of the HIV epidemic will be driven by those fewer individuals who are not on treatment [11]. Therefore, research efforts are still needed to identify, understand, and suggest measures to take to reaching universal coverage of ART among PLHIV.

Barriers to ART access and use among PLHIV in developing countries have been well documented. The most commonly cited barriers to ART use among PLHIV in the developing world are stigma and the distance between place of residence and the health facility [7, 12–15]. Also, lower ART coverage among men than women (68% vs. 71%), and among youth under 30 years than those aged 30–59 years (58% vs. 75%) were observed in KwaZulu–Natal, South Africa, implying that sex and age are important demographic factors playing a role in ART coverage [16]. Furthermore, a recent qualitative systematic review in low- and middle-income countries (LMICs) observed the following reasons for ART nonuse: feeling healthy, low social support, gender norms, difficulties translating intentions into actions, high care–seeking costs, concerns about confidentiality, low quality health services, recommended lifestyle changes, and incomplete knowledge of treatment benefits [7]. Similarly, barriers to ART access in terms of fear to start ART due to drug shortages at health facilities, staffing shortages, and lack of social and economic support were identified in Uganda [14], as well as fear of HIV disclosure, women’s lack of support from male partners, demanding work schedules, and high transport costs [15]. Male gender, younger age, living alone or in households with one to two co-residents, and CD4 count of more than 250 cells per microliter of blood were significantly associated with non-enrollment into free community HIV care in Uganda [17].

In Tanzania, a qualitative study carried out in Iringa Region found significant barriers inhibiting retention to care and treatment for PLHIV. The observed barriers include lack of knowledge and general misconception about treatment, access problems such as difficulties in reaching distant clinics and pervasive poverty that render PLHIV unable to cope with out–of–pocket financial costs associated with HIV treatment, persistent stigmatization of PLHIV, and reliance on alternative healing [18]. The factors perceived to enhance HIV treatment with ART were positive perceptions among the clients of the efficacy of ART, improved ART availability, improved access to care through supplemental aid, and social support [18]. Despite these observations, quantitative and large-scale evaluations are needed to demonstrate statistically representative evidence of conditions and contexts which influence ART use to reach universal coverage of ART among PLHIV.

In assessing the influence of family and social support given to PLHIV, it was observed that while OVC are a well-researched population [19–26], their caregivers have not been prioritized in studies. To date, HIV burden, treatment coverage and health outcomes are not well known among the caregivers. Caregivers play a significant role in the lives and wellbeing of OVC and the entire family, including managing HIV treatment for children LHIV [27]. Research shows that most caregivers have to face economic and food insecurity challenges as a result of additional children to feed and clothe due to AIDS-related orphanhood [28]. Many HIV-affected caregivers do not earn enough income to adequately care for their families [28–30]; they struggle to find safe, affordable childcare, and often corrode their income-generating activities (IGAs) due to caregiving tensions [31]. Since poor health of caregivers results in poor health of their children, strategies to support OVC should target the caregiver-child dyad [32]. For the caregivers LHIV and not on ART, attempts to identify them and ensure that they initiate and continue using ART is critical for their wellbeing and their ability to care for their children. Thus, an improved understanding of the health, social, economic, and psychological challenges faced by the caregivers can inform programs to enhance their ability to be more responsive to their own needs and those of their children [33]. This study explored the extent of and factors associated with ART use among OVC caregivers LHIV in Tanzania. As the country strives for universal health coverage (UHC), knowledge of the factors will alight the high burden sub-populations that should be targeted with additional support so that all PLHIV are ultimately initiated and sustained on ART for better health outcomes and accelerating an end to the HIV epidemic [11]. The government, programs, donors, CSOs and other stakeholders including research communities can benefit from this knowledge to properly plan or advance interventions. Ultimately, with improved policies and programs, ART access and uptake can be scaled among this population and indirectly benefit their children too.

## Methods

### Source of data

Data for this study stem from the community–based, USAID-funded Kizazi Kipya project (2016–2021) in Tanzania, which aims to increase the uptake of age-appropriate HIV-related and other health and social services for improved health and wellbeing by OVC, adolescents and their families. The study used caregivers’ self–reported data collected by Community Case Workers (CCWs) during beneficiary screening and enrollment into the project using the Family and Child Asset Assessment (FCAA) tool from January 2017 to June 2018. CCWs are a government cadre of volunteers trained on the National Integrated Case Management System (NICMS) to provide services to OVC and their caregivers [34]. The USAID Kizazi Kipya project works through this cadre to provide doorstep services to OVC and their caregivers. The project supports the CCWs with a monthly stipend for their work. The caregivers were enrolled into the project if their households met one or more of the 14 household vulnerabilities related to HIV presented in Table 1.

**Table 1:** USAID Kizazi Kipya Project household screening and enrollment criteria

1. Household is headed by child (under 18 years old)	
2. Household is headed by an elderly caregiver (60 years or older)	
3. Household cares for one or more single or double orphan	
4. Caregiver is chronically ill and unable to meet basic needs of children	
5. Caregiver is a drug user	
6. Caregiver or adolescent aged 10–19 years in the household is a sex worker	
7. One or more adolescent girls aged 10–19 years are sexually active	
8. Adolescent girl age 10–19 years in the household is pregnant or has a child of her own	
9. One or more household members are HIV positive	
10. One or more children in the household have tuberculosis	
11. One or more children in the household are severely malnourished	
12. One or more children in the household have been or are abused or at risk for abuse	
13. One or more children are living and or working on the streets, and	
14. One or more children in the household are working in mines.	

Following beneficiary screening and enrollment, the USAID Kizazi Kipya project develops a care plan for each caregiver and their OVC in the household. The project then provides or links caregivers, children and adolescents to services in the areas of health, nutrition, education, child protection, social protection, and economic strengthening. No material support is provided. The project provides psychosocial support, nutrition assessments, counseling and support, referrals and linkages, and care plan monitoring.

To ensure data quality, the USAID Kizazi Kipya project assigned a unique identification number (UID) to each beneficiary enrolled in the project. The project also developed electronic data management systems to track services provided across multiple service delivery points and merge with assessment and demographic data using the UID. These systems have enabled the analysis conducted for this study. Enhanced data validation and logic checks are implemented in the electronic data systems to ensure that appropriate services are provided to the appropriate beneficiaries (e.g. based on age, sex, HIV status etc.) [35].

The project provides supportive supervision to the CCWs through Lead Case Workers (LCWs) and Para Social Workers (PSWs) who offer them mentorship and coaching, and also follow up with them to ensure proper service delivery, timely completeness and correctness of forms and timely submission of data. Each CCW reports to a CSO which also provides supportive supervision to the CCWs through Case Management Coordinators (CMCs). The CSOs provide refresher trainings and conduct monthly meetings with CCWs to discuss performance, challenges, best practices and way forward. Both LCWs and CCWs are supervised by an assigned government social welfare officer at ward level to ensure that services provided and their quality are in accordance with the government guidelines and standards [36, 37]. The USAID Kizazi Kipya project is further described elsewhere [38].

### Study area

This study is based on new beneficiary enrollment data from 39 districts (known locally as councils) in 11 regions of Tanzania where the USAID Kizazi Kipya project conducted beneficiary screening and enrollment activities from January 2017 to June 2018. The regions were: Arusha, Iringa, Katavi, Kigoma, Mara, Mbeya, Morogoro, Ruvuma, Simiyu, Singida, and Tanga.

### Study design

This is a cross–sectional secondary analysis of existing program data of the USAID Kizazi Kipya project. FCAA data were collected during screening and enrollment of beneficiaries into the project. Beneficiaries in households meeting at least one of the enrollment criteria listed in Table 1 and voluntarily consented to participate in the project were enrolled. After enrollment, beneficiaries were followed up by the project over time with a variety of health, education and other social services.

### Study population

This study was based on 74,999 caregivers of OVC who reported that they were living with HIV at the time of screening and enrollment into the USAID Kizazi Kipya project. Caregivers who reported that they were not living with HIV as well as those who did not disclose their HIV status to the project volunteers were excluded. A caregiver is a guardian who has the greatest responsibility for the daily care and rearing of one or more OVC in a household [37]. A caregiver is not necessarily a biological parent of the OVC in that household.

### Variables

The outcome variable for this study is caregivers’ ART use status at enrollment as dichotomized to ‘not on ART (0)’ and ‘on ART (1)’. Caregivers LHIV whose ART status was unknown or not responded, were excluded from the analysis.

Independent variables included were sex, age, marital status, education attained, place of residence (rural or urban), household food security (i.e. whether any household member went 24 h without eating due to lack of food three or more times in the last 4 weeks), health insurance status, mental or physical disability status, and household wealth quintile.

Wealth quintile was constructed using principal component analysis (PCA) of household assets to obtain household socio–economic status [39]. Five wealth quintiles were constructed, ranging from the lowest quintile (Q1) for the poorest households, to the highest quintile (Q5) for the well–off households. The household assets included in the PCA process were; dwelling materials (brick, concrete, cement, aluminium and/or other material), livestock (chicken, goats, cows, and others), transportation assets (bicycle, motorcycle/moped, tractor, motor vehicle, and others), and productive assets (sewing machine, television, couch or sofa, cooking gas, hair dryer, radio, refrigerator, blender, oven, and others).

### Data analysis

The process of data analysis began with exploratory analysis that involved one–way tabulations to obtain distributional features of the data, from which frequency distribution tables were generated. Then cross–tabulations of ART status by each independent variable were performed using the Chi-Square test to assess the significance of the association between ART status and each independent variable.

Multivariable analysis was conducted using multilevel modelling through random–effects logistic regression to identify factors associated with ART use. The value of a multilevel model lies in the fact that it simultaneously considers data hierarchies (e.g. village-level, household-level or individual-level factors), while allowing for non-independence of observations within groups [40, 41]. For the current study, the multilevel model was fitted considering two levels: individual-level and village-level. Higher levels such as district and region were not statistically justified for inclusion (i.e. very low intraclass correlation coefficients [42]), suggesting independence of observations in those levels. The model was chosen to account for clustered structure of the data [43]. Clustering of caregivers was assumed at a village-level. Specifically, we assumed that caregivers LHIV who live in the same village may be correlated with respect to ART use because they may be likely to share the same sources of, barriers to, and enablers of ART use. Independent factors associated with ART use were inferred at a significance level of 5% or less.

Furthermore, the multivariable analysis involved the construction of three models. The first model comprised the entire study population of 74,999 OVC caregivers. The remaining models broke down the study population by sex: the second model was for 54,170 female OVC caregivers, and the third model was for 20,829 male OVC caregivers. These approaches (i.e. multivariable analysis and stratification) played a roles as strategies to address confounding [44–47] among others. Also, stratification by sex provides a deeper understanding of how correlates of ART use differ by gender and inform appropriate interventions that are responsive to the unique needs of each gender [48–50]. For example, one study in Zambia observed that while poverty-related factors hampered ART uptake in women, side effects and social pressure attributable to masculinity did so in men [49]. This would not have been observed without stratification by gender.

## Results

### Background characteristics

As presented in Table 2, this study was based on 74,999 HIV positive OVC caregivers, 96.4% of whom were on ART. These caregivers were from 5532 villages in Tanzania. The average age of the caregivers was 44.4 years (standard deviation [SD] = 11.7) and the majority were female (72.2%). Nearly half (48.7%) of respondents were married, and the majority had attained primary education (78.9%). Nearly one fifth (17.6%) of the caregivers had never attended school. Rural residences accounted for 63.1% of caregivers, and those from food insecure households were 14.6%.

**Table 2:** Frequency distribution of HIV positive caregivers of orphans and vulnerable children in Tanzania, 2018 (*N* = 74,999)

Covariate	Number of HIV positive caregivers (n)	Percent (%)
**Overall**	**74,999**	**100.0**
**ART status**		
Not on ART	2699	3.6
On ART	72,300	96.4
**Caregiver sex**		
Female	54,170	72.2
Male	20,829	27.8
**Age group (in Years)**		
19–29	5883	7.8
30–39	21,401	28.5
40–49	25,643	34.2
50–59	13,665	18.2
60+	8407	11.2
Mean = 44.4, SD = 11.7	–	–
**Marital status**		
Married or living together	36,525	48.7
Divorced or separated	12,447	16.6
Never been married	5336	7.1
Widowed	20,691	27.6
**Education attained**		
Never been to school	13,191	17.6
Primary	59,182	78.9
Secondary or higher	2626	3.5
**Wealth Quintile**		
Lowest (Q1)	19,763	26.4
Second	12,165	16.2
Middle	13,232	17.6
Fourth	14,437	19.3
Highest (Q5)	15,402	20.5
**Place of residence**		
Rural	47,305	63.1
Urban	27,694	36.9
**Household food security status**		
Insecure	10,940	14.6
Secure	64,059	85.4
**Family has health insurance (CHF/TIKA)?**		
No	63,673	84.9
Yes	11,326	15.1
**Mentally or physically disabled?**		
No	73,872	98.5
Yes	1127	1.5

*CHF* Community Health Fund, *TIKA Tiba kwa Kadi*

### ART use by background characteristics

Table 3 presents the percent of caregivers LHIV who were currently on ART at the time of enrollment in the USAID Kizazi Kipya project by their background characteristics. The proportion of the HIV positive caregivers who were on ART varied significantly by some background characteristics. The proportion of caregivers LHIV who were on ART was 95.2% among the oldest (60+ years) as compared to 96.7% among those who were aged 40–49 (*p* < 0.001). ART use was higher among those in marital unions (96.7%) than among those widowed (96.0%) (*p* = 0.001). ART use was 95.6% among caregivers who had never been to school, 96.6% among those who had primary education, and 95.8% among those who had secondary or higher education (*p* < 0.001). ART use also varied by wealth quintile, whereby the lowest level was 95.8% among caregivers in the middle quintile, and the highest level was 97.2% among those who were in the second quintile (*p* < 0.001). ART use was higher in rural areas than in urban areas (96.7% versus 96.0%) (*p* < 0.001). Food security was also a significant dimension of ART use, whereby ART use was higher among caregivers from food secure households (96.6%) than those in food insecure households (95.3%) (*p* < 0.001). ART use was higher among caregivers with health insurance than those without it (96.9% versus 96.3%) (*p* = 0.005). Finally, ART use was as low as 93.4% among caregivers with mental or physical disability and as high as 96.5% among caregivers without any of the disabilities (*p* < 0.001).

**Table 3:** Percent of HIV positive caregivers of orphans and vulnerable children who were currently on ART at enrollment by background characteristics in Tanzania, 2018 (*N* = 74,999)

Covariate	% of HIV positive caregivers currently on ART	*P - value
**Overall**	**96.4**	**–**
**Caregiver sex**		0.348
Female	96.4	
Male	96.3	
**Age group (in Years)**		< 0.001
19–29	96.2	
30–39	96.6	
40–49	96.7	
50–59	96.5	
60+	95.2	
**Marital status**		0.001
Married or living together	96.7	
Divorced or separated	96.4	
Never been married	96.2	
Widowed	96.0	
**Education attained**		< 0.001
Never been to school	95.6	
Primary	96.6	
Secondary or higher	95.8	
**Wealth Quintile**		< 0.001
Lowest (Q1)	96.1	
Second	97.2	
Middle	95.8	
Fourth	96.2	
Highest (Q5)	96.9	
**Place of residence**		< 0.001
Rural	96.7	
Urban	96.0	
**Household food security status**		< 0.001
Insecure	95.3	
Secure	96.6	
**Family has health insurance (CHF/TIKA)?**		0.005
No	96.3	
Yes	96.9	
**Mentally or physically disabled?**		< 0.001
No	96.5	
Yes	93.4	

*p—values are based on Pearson’s Chi–Square test

### Results from multivariable analysis

In Table 4, adjusted odds ratios (OR) and their corresponding 95% confidence intervals (CI) of the factors associated with ART use among caregivers LHIV are presented, accounting for the random effects. Based on this context, the following interpretations are made: In the overall model, age was a significant predictor of ART use only for the age group 40–49 (OR = 1.18, 95% CI 1.00–1.40). The effect of age on ART use was clearer in the stratified analysis, whereby ART use was 30% less likely among the oldest female caregivers than the youngest females (female age 60+: OR = 0.70, 95% CI 0.56–0.87). On the other hand, the likelihood of ART use increased with age among male caregivers (male age 30–39: OR = 1.63, 95% CI 1.13–2.35; male age 40–49: OR = 2.04, 95% CI 1.42–2.92; male age 50–59: OR = 2.14, 95% CI 1.47–3.12; and male age 60+: OR = 1.71, 95% CI 1.16, 95% CI 1.16–2.52).

**Table 4:** Multivariable random–effects logistic regression of factors associated with ART utilization in HIV positive caregivers of orphans and vulnerable children in Tanzania, 2018

Covariate	All (***N*** = 74,999)			Women (***N*** = 54,170)			Men (***N*** = 20,829)		
	Odds ratio (OR)	95% Confidence Interval (CI)		Odds ratio (OR)	95% Confidence Interval (CI)		Odds ratio (OR)	95% Confidence Interval (CI)	
		Lower Limit	Upper Limit		Lower Limit	Upper Limit		Lower Limit	Upper Limit
**Caregiver sex**									
Female	1.00	**–**	**–**	**–**	**–**	**–**	**–**	**–**	**–**
Male	0.92	0.83	1.02	**–**	**–**	**–**	**–**	**–**	**–**
**Age group (in Years)**									
19–29	1.00	**–**	**–**	1.00	**–**	**–**	1.00	**–**	**–**
30–39	1.11	0.94	1.31	1.05	0.87	1.27	**1.63	1.13	2.35
40–49	**1.18	1.00	1.40	1.06	0.88	1.28	***2.04	1.42	2.92
50–59	1.16	0.97	1.39	0.99	0.80	1.22	***2.14	1.47	3.12
60+	0.86	0.71	1.04	**0.70	0.56	0.87	**1.71	1.16	2.52
**Marital status**									
Married or living together	1.00	**–**	**–**	1.00	**–**	**–**	1.00	**–**	**–**
Divorced or separated	0.96	0.85	1.09	0.93	0.81	1.07	1.00	0.77	1.29
Never been married	0.91	0.77	1.08	*0.84	0.70	1.02	1.50	0.87	2.61
Widowed	0.95	0.85	1.06	0.95	0.84	1.08	0.93	0.73	1.17
**Education attained**									
Never been to school	1.00	**–**	**–**	1.00	**–**	**–**	1.00	**–**	**–**
Primary	***1.26	1.13	1.41	***1.27	1.12	1.45	**1.30	1.04	1.62
Secondary or higher	1.09	0.86	1.38	1.12	0.85	1.48	1.08	0.68	1.72
**Wealth Quintile**									
Lowest (Q1)	1.00	**–**	**–**	1.00	**–**	**–**	1.00	**–**	**–**
Second	***1.39	1.20	1.61	**1.31	1.11	1.54	***1.98	1.42	2.78
Middle	**0.84	0.74	0.95	**0.83	0.72	0.96	0.93	0.71	1.21
Fourth	**0.88	0.77	1.00	*0.87	0.75	1.01	1.10	0.86	1.40
Highest (Q5)	0.98	0.85	1.13	0.91	0.77	1.09	*1.26	0.99	1.61
**Place of residence**									
Rural	1.00	**–**	**–**	1.00	**–**	**–**	1.00	**–**	**–**
Urban	***0.70	0.61	0.81	***0.69	0.59	0.80	**0.72	0.56	0.91
**Household food security status**									
Insecure	1.00	**–**	**–**	1.00	**–**	**–**	1.00	**–**	**–**
Secure	***1.29	1.15	1.45	**1.23	1.07	1.41	***1.61	1.28	2.03
**Family has health insurance (CHF/TIKA)?**									
No	1.00	**–**	**–**	1.00	**–**	**–**	1.00	**–**	**–**
Yes	**1.19	1.04	1.36	1.09	0.94	1.28	**1.43	1.11	1.83
**Mentally or physically disabled?**									
No	1.00	**–**	**–**	1.00	**–**	**–**	1.00	**–**	**–**
Yes	***0.58	0.45	0.76	**0.68	0.48	0.97	***0.37	0.23	0.58
	Number of Caregivers = 74,999; Number of Villages = 5532; min = 1, avg. = 13.6, max = 455; Intraclass correlation coefficient = 0.40			Number of Caregivers = 54,170; Number of Villages = 5258; min = 1, avg. = 10.3, max = 358; Intraclass correlation coefficient = 0.40			Number of Caregivers = 20,829; Number of Villages = 4147; min = 1, avg. = 5.0, max = 136; Intraclass correlation coefficient = 0.44		

Significance: ***p < 0.001, **p < 0.050, *p < 0.10

HIV positive caregivers with primary education were 26% more likely to be on ART than those who had never attended school (OR = 1.26, 95% CI 1.13–1.41). This effect was maintained for both female caregivers (OR = 1.27, 95% CI 1.12–1.45) and male caregivers (OR = 1.30, 95% CI 1.04–1.62). Similarly, secondary education or higher was associated with higher likelihood of ART use, but the effect was not statistically significant.

Wealth quintile was associated with ART use in a mixed fashion. Generally, caregivers in higher wealth quintiles were associated with a lower likelihood of ART use than the lowest quintile, with the exception of the second quintile in which caregivers were 39% more likely to be on ART than the lowest quintile (OR = 1.39, 95% CI 1.20–1.61). In the stratified models, women exhibited the same pattern of ART use as the overall group, but in men, the second, fourth and the highest wealth quintiles suggested increased likelihood of ART use, though only the second quintile showed significance (OR = 1.98, 95% CI 1.42–2.78).

Caregivers living in urban areas were 30% less likely to be on ART than their rural counterparts (OR = 0.70, 95% CI 0.61–0.81). This finding was consistent for female caregivers (OR = 0.69, 95% CI 0.59–0.80) and male caregivers (OR = 0.72, 95% CI 0.56–0.91). Caregivers living in food secure households were 29% more likely to be on ART than those in food insecure households (OR = 1.29, 95% CI 1.15–1.45). While the direction and significance of the effect remained, the magnitude was lowest (OR = 1.23, 95% CI 1.07–1.41) in females and highest in males (OR = 1.61, 95% CI 1.28–2.03). Caregivers with health insurance were 19% more likely to be on ART than those without it (OR = 1.19, 95% CI 1.04–1.36). This effect was statistically significant among male caregivers (OR = 1.43, 95% CI 1.11–1.83), but not in female caregivers (OR = 1.09, 95% CI 0.94–1.28). The presence of mental or physical disability was associated with a 42% lower likelihood of being on ART (OR = 0.58, 95% CI 0.45–0.76). The direction and significance of the effect remained, but the magnitude was lowest (32%) in female (OR = 0.68, 95% CI 0.48–0.97) and highest (63%) in male caregivers (OR = 0.37, 95% CI 0.23–0.58).

The intraclass correlation coefficient (ICC) suggested that 40% of the variability in ART use was due to residence of the caregivers in the same village (ICC = 0.40). This was 40 and 44% in female and male caregivers, respectively.

## Discussion

This study assessed the extent of, and factors associated with ART utilization among HIV positive caregivers of OVC in Tanzania. Results showed that 96.4% of all the 74,999 caregivers who reported that they were LHIV were on ART at enrollment. This proportion was high and slightly exceeded the national estimate of 93.6% as the proportion of adults aged 15 years and older LHIV who are on ART [10], which reflects the considerable progress the Government of Tanzania has made in linking PLHIV to treatment services. Although this level of ART use nears universal coverage for people who are aware of their HIV status, the current efforts need to be accelerated because the future of the HIV epidemic and its associated morbidities will be driven by the small segments of the infected populations who are not reached with and sustained on HIV treatment.

In the multivariable analysis, several caregiver characteristics showed significant association with ART use. Caregivers aged 40–49 years were 18% more likely to be on ART than the younger ones in the age group 19–29 years. The higher likelihood of being on ART as age advanced was clearer for men, since men in all higher age groups were significantly more likely to be on ART than those in the youngest age group, 19–29 years. Although this observation was consistent with others in Uganda [17] and South Africa [16], the underlying mechanism was not clear, hence a need for further research. On the other hand, the current study also observed that female HIV positive caregivers age 60+ years were 30% less likely to be on ART than their counterparts in the youngest age group. While this suggests a clear need for targeted strategies to enhance ART uptake among female elderly caregivers, it was also not clear why this was the case. More studies are required to further explore this association.

Caregivers with primary education level were more likely than those who had never been to school to be on ART. This was also the case in both male and female caregivers in the stratified analysis. However, although there was an increased likelihood of ART use among caregivers with secondary education or higher, the increase was not statistically significant. It is possible that people with at least some education are more likely to comprehend the value of ART, thus use it to stay healthy. Also, they may be at a better position to understand the instructions for using ART. A recent systematic review concluded that incomplete knowledge of the benefits ART was a reason for ART nonuse [7]. Another study in Brazil observed late ART initiation due to low education levels [51]. Therefore, encouraging formal education attainment even at the primary level, is likely to improve ART uptake and possibly other health and social services. Education can reduce treatment misconceptions as well as the use of alternative healing [18], which are not scientifically proven to improve health outcomes in PLHIV.

HIV positive caregivers residing in urban areas were less likely than their rural counterparts to be on ART. However, there are many studies showing higher likelihood of ART utilization in urban areas than in rural areas [52–54]. Although the infrastructural, health and social service conditions of rural and urban settings are greatly diverse, with a generally better condition in urban settings than rural ones, especially in low– and middle–income countries [55], confounders such as cultural and behavioral factors associated with rural compared with urban settings may explain these differences in ART uptake. Rural populations have been reported to be more communal than urban populations [56]. This may be one of the likely routes for better social support structures which are key to ART use [7, 55, 57]. While more studies may be needed to explain the mechanisms through which ART use and rural–urban residences are related, overall, there is a need to tailor interventions to the specific needs of each area [58] to ensure that all PLHIV are on treatment.

Food security was a significant determinant of ART use in the HIV positive caregivers, whereby those from food secure households were more likely to be on ART than those from food insecure households. This observation remained significant in the stratified analysis for both female and male caregivers. This observation is consistent with many others which have established that lack of food is a significant barrier to ART utilization and adherence [14, 55, 58–60]. People may be hesitant to start ART if they do not intend to stay on it due to such reasons as lack of food. This suggests that integrating food support into HIV programs may improve coverage of ART in PLHIV, especially those with limited access to sufficient food.

The results showed an increased likelihood of ART use among caregivers with health insurance than those without, especially for male caregivers. It has been observed that health insurance increases the probability of seeking care [61], and plays a substantial role in increasing access to facility–based care [62]. The variability in ART use by health insurance ownership suggests presence of reasons other than costs because ART is free in Tanzania. One’s decision to acquire health insurance may be a function of knowledge and or perceived usefulness, thus a sign of good health-seeking behavior [63]. One possibility for this observation could be that, as caregivers access their insurance-enabled health services, they may also have to disclose their HIV status to the health provider, thus attracting HIV-related counselling and treatment services for their overall wellbeing. Therefore, while expanding the coverage of health insurance is required to facilitate access to health services, this is likely to encourage ART use among PLHIV. This may be a useful strategy, among others, for enhanced ART coverage among caregivers LHIV, especially men. Men are more likely than women to demonstrate poor health behavior, such as late ART initiation [64], thus necessitating targeted support. This is further supported by the results which demonstrate a stronger relationship between health insurance and ART use among men than women. This may be due to less need for insurance among women than men: women generally demonstrate stronger health seeking behavior than men, and combined with the free ART distribution, may be less influenced by insurance to take up ART than their male counterparts.

The current study also found that OVC caregivers LHIV who were physically or mentally disabled were significantly less likely to be on ART than those without the disabilities. These findings held true through the stratified analyses, among both male and female caregivers. Similar studies have linked mental health with HIV treatment uptake [65, 66], but the influence of physical disability is unknown. There may be physical and logistical challenges to ART access among disabled PLHIV, thus a need for programs to target the disabled with interventions to improve ART use among them. It has been noted that physical or mental disabilities attract the attention of non-disabled persons, often triggering prejudices, and discriminatory behaviors. Therefore, the presence of disability and disease can lead nondisabled to avoid contact with the disabled or even to engage in making antisocial comments or actions [67]. This, in most cases, results into self-stigma [68], a phenomenon that has been associated with poor use of ART in PLHIV [7, 14, 15].

Wealth quintile was also a significant predictor of ART use among the OVC caregivers. Those in the second wealth quintile were more likely to be on ART than those in the lowest wealth quintile. This was the case overall, as well as among male and female caregivers. However, higher wealth quintiles (i.e. middle and fourth) were significantly associated with lower likelihoods of ART use in the overall model as well as in women. The middle and fourth wealth quintiles were not significant in the men’s model, but the fifth wealth quintile was associated with higher likelihood of ART use than the lowest quintile. These disparities in ART use between male and female caregivers in the middle through fifth wealth quintiles were not clear, thus a need for further research to clarify the observed relationship. Overall, other studies have observed higher likelihood of ART use with better economic status [7, 14, 57, 58, 69].

Finally, as the intra-class correlation coefficient (ICC) indicated, there was a significant clustering of the HIV positive caregivers at village level, whereby up to 40% of the variability in ART use among them was due to residence in the same village. This indicated that there may be closer social interactions and communication among the caregivers who reside in the same village, thus likely to behave similarly with respect to ART use. This, somehow, can be shaped by the possibility that these people may be receiving ART from the same facility. Also, they may be facing similar environmental obstacles or enablers to ART use, such as distance to facility, which may further explain the relationship. It has been observed that social support from the family, friends or peers (which is more likely among those in the same vicinity) is an important dimension of ART use [7, 55, 57, 70].

### Limitations

The data were self-reported by the caregivers, allowing for the possibility of recall or information bias in the data. To minimize this, the CCWs who conducted the screening and enrollment exercise were trained to probe the caregivers for accuracy in responding to the questions.

## Conclusions

ART use among OVC caregivers who report their HIV positive status in Tanzania is high (96.4%) and predicted by age, formal education attainment, place of residence, food security, health insurance, mental or physical disability, and wealth status. Since a few caregivers reporting their HIV positive status remain unreached, to achieve universal ART coverage in this population, interventions and policy guidelines should address the observed demand–side barriers such as older age (60 years and above) for female caregivers, and below 30 years for male caregivers; education level, particularly those who have never been to school as they may be having difficulties comprehending ART value for their health. Health providers should be trained to communicate about ART use to low-education patients to improve ART initiation among them. Other barriers that may be integrated in HIV programming activities include urban residences, food insecurity, lack of health insurance particularly for men, and mental or physical disability. Although there may be a few caregivers LHIV who are disabled, as well as those unlikely to be on ART in other subgroups, it is equally crucial to understand this and plan how to reach them because global emphasis to ending the HIV/AIDS epidemic requires leaving no one behind – testing and treating all [71]. This implies that, high burden sub-populations represent the ultimate future of the HIV epidemic, unless targeted efforts are timely made to ensure universal coverage of care and treatment services.

## Data Availability

This study is based on data from the USAID Kizazi Kipya project (2016–2021) in Tanzania. Pact Tanzania is the prime organization implementing the project, hence owns the data. The datasets analyzed during the current study are not publicly available due to confidentiality restrictions pertaining to records of the project beneficiaries, but are available from Pact Tanzania on reasonable request.

## Abbreviations

AIDS: Acquired immunodeficiency syndrome
ART: Antiretroviral therapy
CD4: Cluster of differentiation 4
CHF: Community health fund
CI: Confidence interval
FCAA: Family and child asset assessment
HIV: Human immunodeficiency virus
ICC: Intra-class correlation coefficient
LHIV: Living with HIV
LMICs: Low- and middle-income countries
MRCC: Medical Research Coordinating Committee
MUAC: Mid-upper arm circumference
NIMR: National Institute for Medical Research
OR: Adjusted odds ratio
OVC: Orphans and vulnerable children
PCA: Principal component analysis
PLHIV: People living with HIV
SD: Standard deviation
TIKA: *Tiba kwa Kadi*
UHC: Universal health coverage
UNAIDS: Joint United Nations Programme on HIV/AIDS
USAID: United States Agency for International Development
